# Progress in Phototherapy

**Published:** 1902-11-22

**Authors:** 


### Progress in Phototherapy.

### X-Rays Etc.

The advantages and disadvantages of the Finsen Light and the X-ray treatments are contrasted in a paper founded upon the experience of Malcolm Morris and Dore.[1] They hold that judiciously combined, where necessary, with other means the Finsen light holds first place in the treatment of lupus in the majority of cases. It gives the best cosmetic results and is very reliable, but they are not satisfied as to the permanence of the cure. There is great difficulty in ascertaining with certainty that all the disease is eradicated, and repeated treatment is often found necessary. However, this repetition does no harm to the tissues, and indeed scars are benefited. During treatment disease becomes evident which was not seen before. There are undoubted disadvantages in the treatment—the length of time it takes and the tediousness of it, the small area covered, the expense of the apparatus and the large staff of attendants required are all against it. With regard to the apparatus they still prefer the original lamp of Finsen. The new French lamp (Lortet and Genoud's) has some advantages, but this and the Danish lamp with water-cooled iron electrodes, are too superficial in their action. They are useful for superficial disease or to prepare the ground for the deeper treatment with the original lamp. The X-rays are a useful adjunct to, but not a substitute for the light. The results with the light are more permanent. There is less control with the X-rays, and the scar tends to contract more and to become more discoloured, denser, less supple and tenser. The X-rays differ from light, among other things, in that they show a selective action on diseased tissues, but affect also normal skin—pressure is not required—cause loss of hair, and are not bactericidal. The advantages are that under their influence ulcerations will heal rapidly, and mucous membranes can be reached which are inaccessible to light. In working upon mucous membranes it is advisable to focus the rays directly upon the part, not for instance through the skin and cartilage of the nose, but through a speculum. With regard to reactions : in light treatment the therapeutic effect is much greater when a reaction is obtained. This reaction consists of an inflammation with the formation of blebs and serous crusts, more or less superficial. With the X-rays things are different, the reaction varies from slight transient erythema to necrosis of long duration. This reaction may come on soon or be delayed for some days.

The factors to be taken into consideration are as follow :—(1) Duration of exposure; (2) interval between exposures ; (3) distance of tube from patient; (4) kind of tube employed; (5) degree of vacuum; (6) strength of current; (7) nature of coil and interruptor, etc. ; (8) peculiarities of patient's skin. Hyperæmic cases react quickest to both forms of radiant energy.

According to Lancashire [2] the best results are to be obtained by short applications, 10 to 15 minutes, given at a distance of 8 or 9 inches from the patient : the reaction may take 14 days to develop in its entirety. There is some immunity from recurrence of the reaction after a dermatitis has healed. He advises the use of a soft tube, cleansing the surface of the spot to be treated from débris. During the first week exposures should be given daily, then three times a week, only doing a small part at a time. He has found it useful in hypertrichosis, but according to Schiff the treatment should be at intervals extending over a period of 18 months. This loss of hair is one of a sequence of events, which, according to Freund are as follow :—(1) pigmentation, (2) erythema, (3) inflammatory exudation, (4) loosening of hairs, (5) loss of pigment in dark hairs, (6) itching and feeling of tension. He has used it with success in coccogenic sycosis ; the reaction being inimical to the pus organisms. In lupus vulgaris, good results can be obtained comparatively painlessly, and so excessive reaction should be avoided. This should be done by comparatively long intervals to allow of subsidence of reaction. Old scars become more flexible, which is of use in cases of hide-bound lupus. He recommends this treatment in (1) cases of disease of prominent parts, such as the face, where the mischief is too extensive for the Finsen treatment; (2) some ulcerated cases ; (3) cases associated with unsightly scars ; (4) some cases of disease in mucous membranes. In rodent ulcer he reports two cures after 4 and 6 months respectively. He thinks the treatment should be continued after the cure to prevent relapse.

With regard to hypertrichosis, at the Vienna Dermatological Society [3] the suggestion was made that the depilatory powers of X-rays were due to the ultra-violet light producing hyperæmia and finally a paresis of the blood-vessels. In 40 cases of Jutassy's and 7 under the care of Schiff and Freund, sufficient time has now elapsed for them definitely to state that the hair will not grow again after removal for hypertrichosis with X-rays. With regard to the reaction to X-ray treatment, Kummel denies that dermatitis has any part in the cure. He thinks the beneficial effect dependent upon some peculiar action directly upon the diseased tissues. But C. L. Leonard thinks the results are due to the static electric field in the vicinity of the tube. Rinehart, however, refutes this by the statement that skin can be subjected to static electricity of greater intensity than ordinarily used in X-ray work without effect. The latter investigator and Thompson [4] agree with many in maintaining that the best effects upon the skin are produced by low vacuum tubes. Albers, in 1900, cautioned against inflammatory reaction and Kummel advised avoiding any irritation of the skin, as also did Schiff. Rinehart states, on the other hand, that he cannot obtain any benefit in any case of the kind without an inflammatory reaction.

[1] Brit. Med. Jour , May 31, 1902. [2] Ibid. [3] Amer. Jour. Med. Sci., July 1902.

*(To be continued.)*

